# A method to validate viral copy-number assay involving a hybrid amplicon and duplex droplet digital PCR

**DOI:** 10.1016/j.omtm.2025.101483

**Published:** 2025-04-30

**Authors:** Raymond Wu, Frank Luh, Soo-Mi Kweon, Yun Yen

**Affiliations:** 1 Sino-American Cancer Foundation, 668 Arrow Grand Circle, Covina, CA 91722, USA; 2 Carrigent Inc., 668 Arrow Grand Circle, Suite #205, Covina, CA 91722, USA

**Keywords:** viral copy number, quality control, reference standard, ddPCR, assay development, cell and gene therapy

## Abstract

Viral copy-number (VCN) assay is a powerful, effective method to quantify toxicity, cellular kinetics, and durability of virus-modified cell therapy products. The qualification and validation of assay requires reference control. Traditionally, plasmids and cell lines are used as reference controls, but development and qualification of those controls require considerable time and resources. We propose a reference synthetic DNA fragment containing amplicons of woodchuck hepatitis virus posttranscriptional regulatory element (*WPRE*) and ribonuclease P protein subunit p30 (*RPP30*), connected by HindIII restriction enzyme cutting site, as a useful tool to qualify and validate duplex droplet digital PCR (ddPCR) assays for VCN. Using this hybrid amplicon, we qualified the duplex WPRE/RPP30 ddPCR assay by determining range of quantification, precision, bias, and robustness of the assay. The varying amount of input DNA showed upper limit, lower limit, and linearity of the assay. Coefficient of variation (CV) and % recovery showed assay precision and accuracy, respectively. Furthermore, the hybrid amplicon was used to determine assay robustness with potential conditions of variability. The hybrid amplicon was a comparable alternative to cell reference standards for validating VCN assay. In conclusion, *WPRE-RPP30* hybrid amplicon can be used as a routine quality control measure to validate digital PCR assays.

## Graphical abstract

**Figure:**
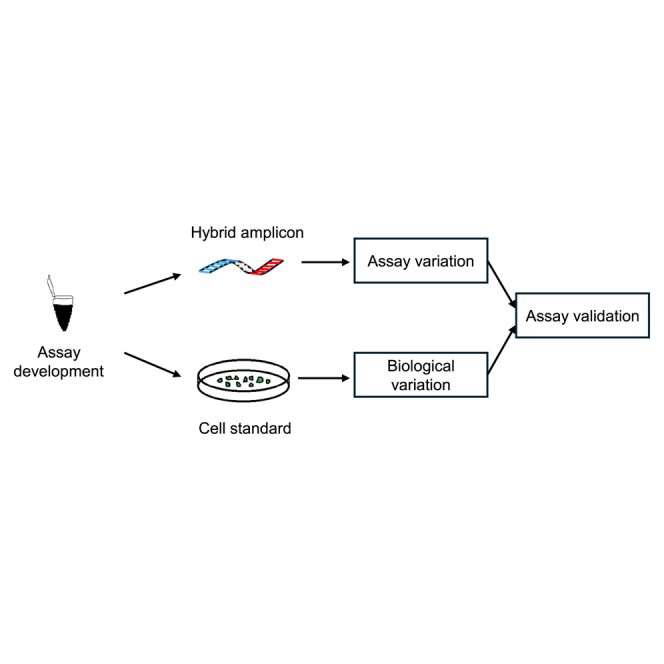

## Teaser

During viral copy-number assay development, positive reference control is critical to ensure validity and reliability. This manuscript describes the use of hybrid amplicon method as reference material as a quicker alternative for assay development. The hybrid amplicons and cell reference are excellent reference materials for validation of VCN assay.

## Introduction

Digital polymerase chain reaction (PCR) technology offers several advantages over traditional PCR, including analytical sensitivity and improved precision.^1^^,^^2^ Digital PCR (dPCR) minimizes the variability of standard PCR reactions by partitioning PCR reactions in smaller volumes.^3^ Partitioning in digital PCR results in thousands of data points that provide more accurate results at the end of the amplification process, making dPCR analysis suitable for detection of small fold-change differences. In addition, dPCR does not require reference standards to determine the concentration of the original sample. The number of positive and negative partitions are quantitated in a Poisson distribution to estimate the absolute amount of target molecules in the original sample. The frequency of positive and negative droplets, according to Poisson distribution, determines the concentration of target molecules in each sample. This unique facet of digital PCR allows an absolute quantification of target molecules.^4^

One limitation to quantifying the sample concentrations in dPCR reactions is the requirement of knowing the exact volume of partition. Currently, there are two major types of digital PCR platforms: microfluidic-based and droplet-based PCR. In microfluidic technology, PCR reactions are performed in small microfluidic chambers. In contrast, in the droplet digital PCR (ddPCR) technology, PCR is performed in oil emulsion droplets. After PCR reaction is complete, each droplet is analyzed for fluorescent intensity in droplets containing positive target molecules versus those that do not. The volume of each partition in microfluidic technology is theoretically constant since the chamber volume is unchanged. However, errors can still arise from bubbles trapped in the chambers during high temperatures in PCR cycles.^5^ Another limitation of ddPCR is that the volume of partitions in each droplet may fluctuate. Although commercial robotic droplet generators have since been designed to minimize this source of variation,^6^ the accuracy and precision of ddPCR are still highly dependent on each volume of partition and consistency.^7^ High-resolution microscopy and optical profilometers are commonly required to accurately measure the volumes of droplets.^6^ However, these instruments are not always available in every lab setting. Furthermore, standard operation procedures may differ between labs.

One strategy to overcome volume partition challenges associated with dPCR is adoption of commercially available reference material. One traditional type of reference material used in PCR assay is plasmid DNAs.^8^ However, disadvantages of using plasmid DNA include the requirement to isolate high-quality plasmids and lot-to-lot variability. In addition, the presence of supercoiled DNA in plasmid samples underestimate the DNA concentration in ddPCR assays.^9^^,^^10^ Another type of reference material commonly used in PCR-based assays is synthetic DNA. In these contexts, the DNA is spiked into next-generation sequencing (NGS) assays for qualitative positive control^11^ to qualify assay performance. In addition to qualifications, using synthetic DNA as reference material may be applied to estimate % recovery of the assay. Since washes are not involved in PCR assays, % recovery corresponds to the volume and efficiency of reaction. In dPCR assays, since PCR efficiency is not applicable due to partitioning, % recovery can be correlated to the volume of total partitions excluding the dead volume. For microfluidic-based dPCR, % recovery corresponds to the total volume of partition without the volume of bubbles. Conversely, for droplet-based dPCR, % recovery corresponds to the total volume of droplets. The dead volume for ddPCR method is the total volume of droplets subtracted from the total volume of test reaction.

Here, we developed a quantitative method to validate ddPCR reaction that can be applied for multiple contexts, including qualification of instrument, operator performance, quality control checks, and inter-run precision and accuracy. This method utilizes a reference material to quantitate the performance of the ddPCR runs. We demonstrated the utility of this approach with viral copy-number (VCN) assay, a common method used to quantify stable gene transfer into target cell populations. The assay detects the absolute copy number of viral gene and absolute copy number of host cell gene. To perform this assay, we synthesized a reference hybrid amplicon DNA that links the amplicons of viral gene *WPRE* to reference gene *RPP30* (referred as *WPRE*-*RPP30*). To explore linkage versus non-linkage use, the amplicons were connected by a restriction enzyme HindIII. Using this system, we were able to amplify *WPRE* and *RPP30* equally, resulting in the same % recovery between *WPRE* and *RPP30*. We were able to determine the stability of *WPRE* and *RPP30* amplicons during restriction digestion and other conditions that would normally contribute to errors in traditional VCN assays. In contrast to the traditional approach, this approach does not require extensive work to make cell lines or plasmids or qualify them for use. We advocate adopting this approach to validate the accuracy of dPCR instruments, PCR reagents, and operator performance for routine use in cell and gene therapy.

## Results

### Unreliability of a single gene reference DNA in a quantitative droplet digital PCR assay

To check the accuracy of ddPCR assay, we used double-stranded synthetic DNA representing *WPRE* sequence from Integrated DNA Technologies (IDT). If the PCR reaction was accurate, the results from ddPCR instrument would match the input copy number. However, % recovery decreased as the input copy number of *WPRE* DNA increased (Table 1). On the other hand, the error (%CV) increased as the number of input copy number decreased. As the % recovery increased to 92%, %CV increased to 18%. Therefore, the optimal input copy number seems to be 1,640 input copy number. As the input copy number increased by 10-fold to 16,400, the % recovery decreased by 10%. Therefore, it is unclear whether the lower value in % recovery is the result of the inefficiency in PCR reaction, incorrect concentration of stock DNA, or operator error in pipetting. It also suggests that the use of a single gene reference DNA is not a quantitative positive control for ddPCR assays.

**Table 1:** Percent recovery of input copy number using *WPRE* DNA

Input copy number	Output copy number in 20 μL			Average output copy number	% average recovery	%CV
	Replicate 1	Replicate 2	Replicate 3			
164,600	111,708.7	10,7415.3	97,193.7	105,439.3	64.1	7.07
16,460	12,370.3	12,206.4	12,071.6	12,216.1	74.2	1.2
1,646	1,440.6	1,251.9	1,357.3	1,349.9	82.0	7.0
164.6	134.6	136.4	183.1	151.3	92.1	18.1
16.46	9.2	30.7	16.9	18.9	115.1	57.4
1.646	11.6	8.2	2.3	7.4	450.2	63.0

*WPRE* DNA was used as a reference material to check for accuracy of the assay. The reference material was serially diluted in triplicates, and copy number of *WPRE* was determined using WPRE ddPCR assay. Input copy number is the number of *WPRE* DNA added to each reaction. Output copy number is the number of copy number generated by QX200.

### Construction of a hybrid amplicon *WPRE*-*RPP30* for reference material

**Figure 1:**
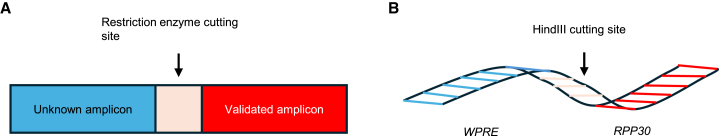
A schematic diagram of synthetic DNA used as reference materials for the validation of viral copy number assay in droplet digital PCR (A) Synthetic DNA can be constructed either through commercially available methods or a combination of PCR and ligation methods. The unknown genetic element can be any endogenous genes or exogenously incorporated genetic elements. Validated genetic element can be any genetic element validated to have a known number of copy numbers in target cells. In the proof-of-principal study, (B) *WPRE* (woodchuck hepatitis virus posttranscriptional element) was used as an unknown genetic element, and *RPP30* was used as a validated genetic element. There are always two copies of *RPP30* in the human genome. Restriction enzymes of choice should not cut both unknown and validated genetic elements.

To eliminate any potential source of error, the reference DNA that links the amplicon of unknown gene to the amplicon of the validated gene was incorporated as reference material. A similar approach comparing alcohol dehydrogenase genes in linearized plasmids has been reported in literature and demonstrated the advantage of linked DNA for duplex dPCR assays.^12^ Plasmids as reference materials have potential drawbacks due to the potential contamination by impurities during plasmid isolation and incomplete digestion of plasmids.^9^^,^^10^ Since the assay for the control gene has been validated, putting the amplicon of the validated gene in the control DNA linked to unknown gene allows an equal comparison between the unknown and validated genes. Regardless of PCR conditions, instrument status, or operator error, the reference material should produce a one-to-one copy of unknown and reference amplicons (Figure 1A). An unknown amplicon can be any amplicon of a gene that needs to be measured experimentally, and validated reference amplicon can be any amplicon of a gene that has been validated. The two amplicons can be connected by the restriction enzyme that would not cut the target amplicons.

In the current VCN assay, the hybrid reference DNA is constructed from *WPRE* amplicon and *RPP30* amplicon connected with HindIII restriction enzyme cutting site. *WPRE*, woodchuck hepatitis virus posttranscriptional regulatory element (WPRE), is commonly found in retroviral vectors to enhance gene expression.^13^ When retrovirus is transduced in mammalian cells, one copy of *WPRE* per virus is inserted in virus-transduced cells. By performing PCR amplification, the number of viruses inserted in each cell can be measured. On the other hand, *RPP30* is a housekeeping gene known to have copy numbers like other reference genes in healthy diploid cells. TCGA copy-number data analysis indicates no significant amplification was observed in the gene region of *RPP30* in many cancer types. In addition, the copy number of *RPP30* and those of other reference genes in CAR-T cells across different cancer types are comparable.^14^ This suggests that *RPP30* can be used in VCN assays involving CAR-T cells. A validated ddPCR assay for *RPP30* was chosen to measure the copy number of *RPP30*. The *WPRE* amplicon has also been published before.^15^ As required by the minimum information for the publication of real-time quantitative PCR experiments (MIQE) guidelines, MIQE sequences are provided for all PCR assays in certificate of analysis.^16^ MIQE sequence was used as an amplicon for *RPP30*. The new *WPRE-RPP30* hybrid amplicon was synthesized by connecting *WPRE* amplicon with *RPP30* amplicon by HindIII restriction enzyme (Figure 1B).

### Evaluation of duplex viral copy-number assay using hybrid amplicon *WPRE-RPP30*

**Figure 2:**
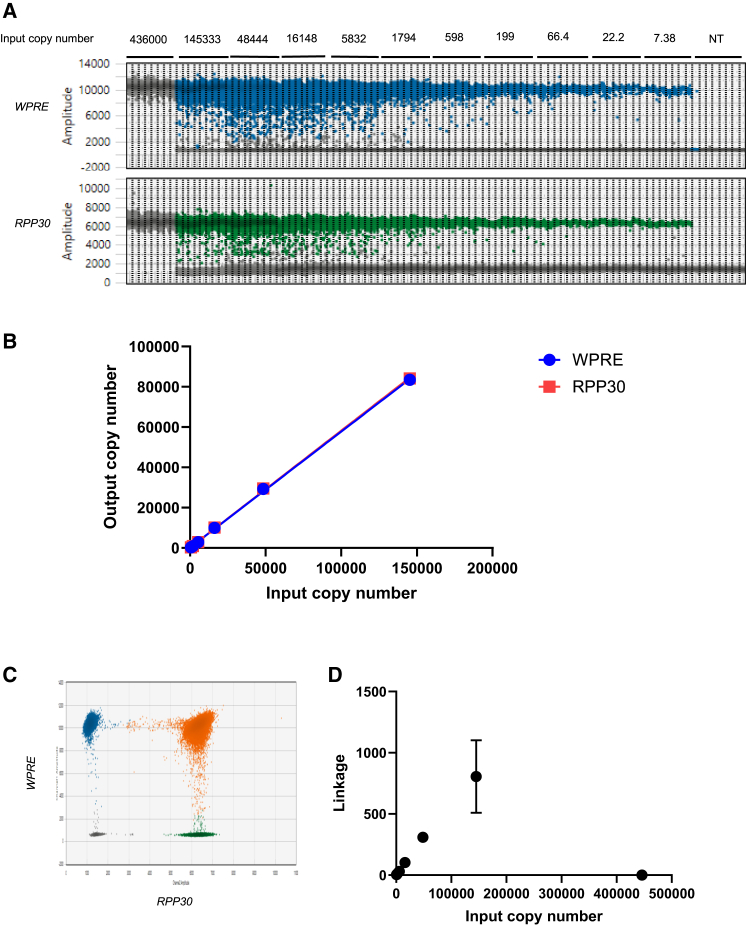
Representative ddPCR plots of *WPRE-RPP30* hybrid amplicons at different input copy numbers A total of 20,000 droplets were captured. DNA was serially diluted 3-fold, and ddPCR reactions were performed. The DNA partition reached saturation at 145000 input copy number, where no empty droplets were detected. (A) 1D representation of titration of *WPRE*-*RPP30* amplicon. The top panel contains positive and negative droplets containing *WPRE* amplicons. The bottom panel contains positive and negative droplets containing *RPP30* amplicons. Each condition was performed in eight replicates. (B) Linearity of the quantification of *WPRE* and *RPP30* amplicons from the hybrid amplicons with *WPRE*/RPP30 duplex ddPCR assay. The input copy number and output copy number of *WPRE* and *RPP30* were plotted, and linear regression was analyzed in GraphPad Prism. R-squared values are 0.9996 for both *WPRE* and *RPP30*. (C) A representative image of 2D plot of droplets containing *WPRE*+RPP30+ amplicon (depicted in orange), singlet amplicons, *WPRE*+*RPP30*− (depicted in blue), *WPRE*-*RPP30*+ (depicted in green), and *WPRE*-*RPP30*− (depicted in gray). (D) Linkage data vs. different input copy number of *WPRE*-*RPP30* hybrid amplicon. The mean values of eight replicates were plotted with standard deviation as errors in GraphPad Prism.

Using *WPRE-RPP30* hybrid amplicon, the duplex VCN assay was evaluated for how amplicons are distributed in droplets. The *WPRE-RPP30* DNA was diluted 3-fold serially from 4.36 × 10^5^ to 7.38 copies to determine how amplicons are distributed at different input copy numbers (Figure 2A). *WPRE-RPP30* DNA occupied all droplets in 4.36 × 10^5^ input copy number. Without empty droplets, quantification was no longer possible, suggesting that quantification reached saturation point at these input copy numbers. The highest quantifiable copy number in our study was 1.45 × 10^5^. This copy number is considered upper limit of quantification (ULOQ). At this input copy number, some droplets are empty, making quantification possible. Positive droplets were detected in all eight replicates for the input copy number of 7.38, making the range of quantification 1.45 × 10^5^ to 7.38. For this range, the input and output copy numbers linearly correlated up to R-squared values of 0.9996 (Figure 2B).

To understand how restriction enzyme cuts the DNA during the preparation, distribution of *WPRE-RPP30* amplicons in the droplets was observed in the 2D representation (Figure 2C). The restriction enzyme HindIII was added to the reaction tube prior to droplet generation. If the cut by HindIII occurred only in the PCR reaction, all positive droplets should contain both *WPRE* and *RPP30* amplicons. However, the results showed that positive droplets contained both amplicons (depicted in orange dots in Figure 2C) as well as single amplicons (depicted in blue and green dots in Figure 2C). The results revealed that HindIII activity occurred spontaneously even before the droplet generation and PCR reaction. Therefore, the hybrid amplicon *WPRE-RPP30* can be used to evaluate the target DNA distribution in droplets and restrict enzyme activity on target molecules.

To determine how restriction enzymes cut the amplicons, linkage data were plotted against different input copy numbers. For the same enzyme amount, the linkage data increased as the input copy number increased. The linkage data were not available at the highest input copy number of 48,444 because of the saturation of all droplets with the target amplicons (Figure 2D). At that concentration, quantification with Poisson distribution is not possible. At the input copy number of 145,000, linkage values varied with a %CV of 36% (Figure 2D). The data suggest that the cutting efficiency of HindIII decreased when the input copy number reached 145,000.

### Efficiency of recovery using hybrid amplicon *WPRE-RPP30*

To eliminate any potential error due to limitation in PCR efficiency or accuracy, % recovery was measured using hybrid amplicon *WPRE-RPP30*. Since the recovery of *WPRE* DNA was not consistent, *RPP30* was added as a reference to rule out any potential error. The *RPP30* assay was validated by the commercial vendor. Therefore, comparison between the absolute quantification of *WPRE* and *RPP30* should eliminate any chance of error during PCR. The % recovery values between *WPRE* and *RPP30* from the hybrid *WPRE-RPP30* amplicon scored similarly across different DNA input copy numbers (Table 2). The hybrid *WPRE-RPP30* amplicon produced approximately 50%–70% recovery of *WPRE* and *RPP30*. In the lowest input copy numbers 22.15 and 4.36, the % recovery values between *WPRE* and *RPP30* were over 100% due to inaccuracy in measuring very low input copy number. The percentage recovery significantly dropped when the input copy number was below 598.08 (Table 2).

**Table 2:** % recovery of *WPRE* and *RPP30* amplicons

Input copy number	Average *WPRE* output copy number (%CV)	Average % recovery (*WPRE*)	Average *RPP30* output copy number (%CV)	Average % recovery (*RPP30*)
145,333.33	83,473.78 (3.4)	57.44	84,149.20 (3.27)	57.90
48,444.44	29,294.24 (2.34)	60.47	29,531.27 (2.39)	60.96
16,148.15	9,999.46 (7.8)	61.92	10,108.21 (7.81)	62.60
5,382.71	2,846.87 (2.46)	52.89	2,864.82 (2.88)	52.89
1,794.23	891.12 (4.87)	49.67	920.63 (5.16)	51.31
598.08	291.82 (9.57)	48.79	309.53 (10.19)	51.75
199.36	105.10 (13.69)	52.72	117.78 (12.39)	59.08
66.45	51.17 (9.2)	77.01	59.21 (14.44)	89.10
22.15	27.04 (20.95)	122.06	28.40 (12.83)	128.23
7.38	26.24 (16.40)	355.51	28.91 (15.39)	391.77

The first column indicates the input copy number. The output copy number is the absolute copy number given by the QX200 droplet reader. Average and %CV values were calculated from eight replicates.

### Accuracy check of duplex viral copy-number assay using hybrid amplicon *WPRE-RPP30*

Although the % recovery values for both amplicons did not reach one hundred percent because of dead volume, the final assay measurement for the VCN assay is *WPRE* copy number normalized to *RPP30* copy number. Therefore, even though % recovery is not one hundred percent, viral copy number can still be accurate as long as % recovery of *WPRE* and *RPP30* are equivalent. As expected, the copy number variation (CNV) values from WPRE/RPP30 duplex assay were close to two for input copy number values from 1.45 × 10^5^ to 7.38. For input copy numbers from 1.45 × 10^5^ to 1,794.23, the CNV values were over 1.9, and %CV values were less than 2% (Table 3). However, at these input copy numbers, a significant number of droplets with lower fluorescent values, often referred to as rain in ddPCR, was observed. These droplets did not affect the %CV of assay at these input copy numbers because the fluorescent separation between positive and negative droplets was high. Precision and accuracy may be lower for input copy numbers lower than 598.08 copy numbers because CNV values started to vary significantly for lower input numbers (Table 3). The results suggest our duplex *WPRE/RPP30* VCN assay is an accurate method to quantify copy number of integrated viral genomes into the host cell at copy number levels ranging from 1.45 × 10^5^ to 7.38. The optimal range of input copy numbers with accuracy and precision for this WPRE/RPP30 assay seemed to be from 1,000 to 10,000 input copy numbers because the error started to increase below 1,000, and rain effect was noted starting at 10,000 input copy numbers (Figure 2A).

**Table 3:** Copy number variation (CNV) of *WPRE* amplicon normalized to *RPP30* amplicon at different input copy numbers

Input copy number	Average copy number variation (CNV)	%CV
145,333.33	1.98	1.29
48,444.44	1.98	1.31
16,148.15	1.98	1.05
5,382.71	1.99	1.70
1,794.23	1.94	1.66
598.08	1.89	4.21
199.36	1.78	6.83
66.45	1.74	9.32
22.15	1.90	15.64
7.38	1.81	4.38

Copy number variation was calculated by copy number of WPRE/copy number of RPP30) × 2.

### Robustness study of VCN assay using hybrid amplicon *WPRE-RPP30*

To determine the accuracy of the assay under potential variable conditions, a robustness study was designed with the hybrid amplicon *WPRE-RPP30*. Three different conditions were tested: (1) presence or absence of restriction enzyme HindIII, (2) input copy number, and (3) preincubation time of restriction enzyme. The presence or absence of restriction enzyme is one opportunity for variability because the operator may forget to add the enzyme or add incorrect concentrations of enzyme. The input copy number could also vary due to pipetting or calculation error. The preincubation time could vary because the droplet generation reaction may not start due to unforeseen reasons. Three input copy numbers were chosen based on their relevancy to the VCN assay in cells; 48,444 and 5,382 input copy number values are relevant to the VCN assay of cells because 48,444 cells of VCN of two would yield the genomic DNA (gDNA) of 290 ng (6 pg × 48,444 cells), and 5,382 cells would yield the gDNA of 32 ng (6 pg × 5,382 cells). It is known that a typical mammalian cell contains 6 pg of gDNA.^17^

**Figure 3:**
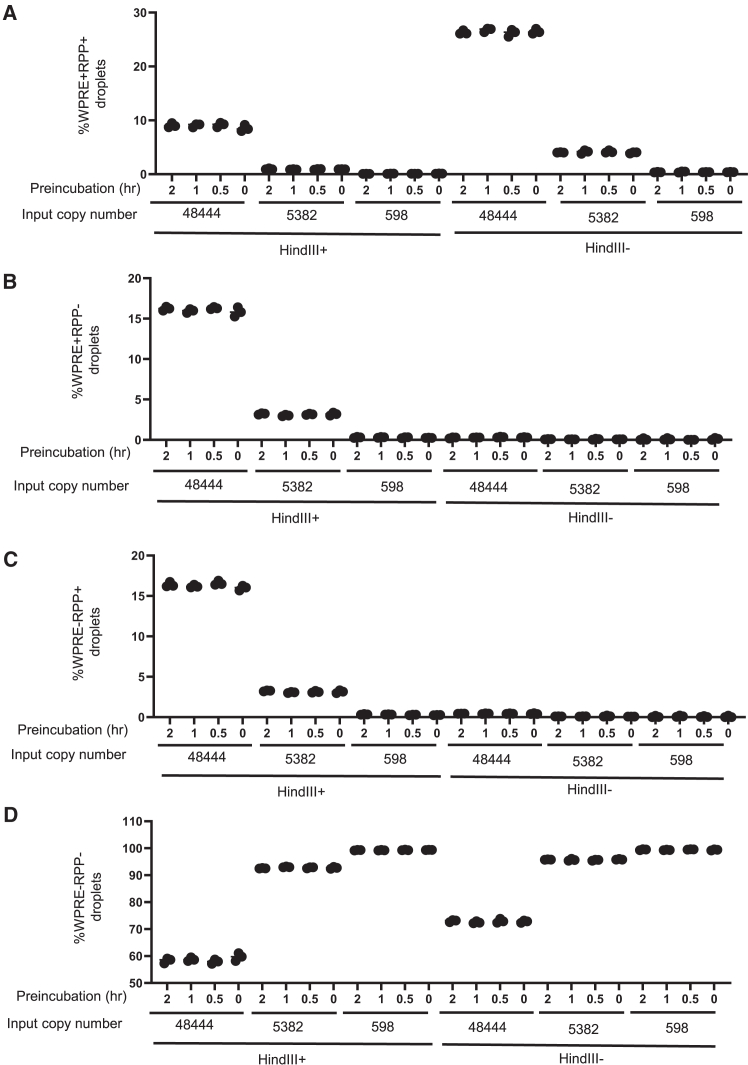
Effect of HindIII on the amplicon distribution in the droplets The hybrid amplicons at different input copy numbers were amplified in *WPRE* and RPP30 ddPCR assays with or without the enzymes. The reactions were preincubated at room temperature at different time points before droplets were generated in the AutoDG instrument. Each condition was performed in triplicates. The mean values of triplicates with standard deviation as errors were plotted. The total numbers of accepted droplets were consistently close to 20,000. (A) The frequency of droplets containing *WPRE*+*RPP30*+ amplicons in total accepted droplets. (B and C) The frequency of droplets containing single amplicons in total accepted droplets. (D) The frequency of empty droplets.

The results revealed preincubating restriction enzyme up to 2 h did not affect the accuracy of the VCN assay. Without HindIII enzyme, the percentage of droplets containing both amplicons was approximately 26% for target input copy number of 48,444 but that number decreased to approximately 9% for the same input copy number when HindIII was added (Figure 3A). Without HindIII enzyme, approximately 0.3% of droplets contained only single amplicons for the input copy number of 48,444. For input copy numbers of 5,382 and 598, the percentage of droplets with single amplicons was below 0.1% without HindIII (Figures 3B and 3C). With HindIII enzyme, the percentage of droplets with single amplicons increased to approximately 16% and 3% for input copy numbers of 48,444 and 5,382, respectively (Figures 3B and 3C). At the same time, HindIII enzyme decreased the percentage of droplets containing both amplicons from 26% to 9% with the difference of 17% (Figure 3A).

These results demonstrate that HindIII cut the hybrid amplicons efficiently by splitting the amplicons into equal numbers. The 17% loss of droplets containing both amplicons led to a 16% gain of single amplicons, indicating HindIII cut both amplicons without degrading both target amplicons. It also indicated the stability of both amplicons after HindIII digestion for at least 2 h at room temperature.

### Restriction digestion decreased the formation of rain but did not affect the accuracy of copy-number quantification

**Figure 4:**
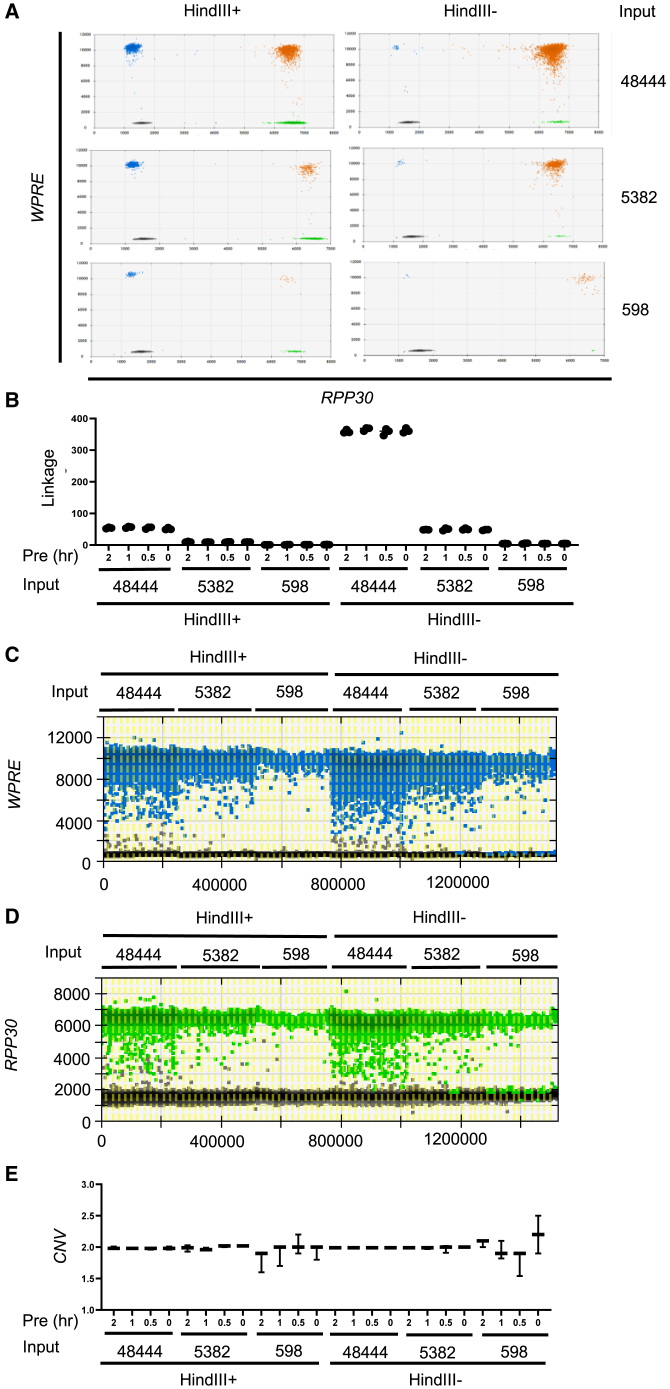
The effect of restriction digestion on the linkage of the hybrid amplicon and potential error (A) Representative pictures of 2D plots of hybrid amplicons in ddPCR assays with or without HindIII. *x* axis represents *WPRE* amplicon, and *y* axis represents *RPP30* amplicon. Orange dots represent the droplets with both amplicons. Blue dots represent the droplets with *WPRE* single amplicon, and green dots represent the droplets with *RPP30* single amplicon. (B) Linkage analysis of the hybrid amplicon with or without HindIII. (C) 1D plot of *WPRE* fluorescence in droplets with or without HindIII. (D) 1D plot of *RPP30* fluorescence in droplets with or without HindIII. (E) Copy number of *WPRE* normalized to *RPP30* with or without HindIII. The bars represent the variation of CNV values of three replicates.

In ddPCR assays, additional use of restriction enzymes is common if more than 60 ng of gDNA is used. Restriction enzyme allows fragmentation of DNA at specific sites to reduce unnecessary shearing of DNA at the target genes due to the mechanical stress. In addition, restriction enzyme can be used to study linkage analysis of the two pieces of connected DNA. The results showed that the presence of HindIII decreased the percentage of WPRE+RPP30+ droplets (orange droplets) and increased the droplets with single amplicons (blue and green droplets) (Figure 4A), suggesting that HindIII cut the two target amplicons. The linkage score of WPRE-RPP30 DNA also decreased from approximately 350 to 60 (Figure 4B), suggesting that this approach allowed the study of linked DNA. The presence of HindIII reduced the rain, a phenomenon that occurred due to the presence of droplets with lower fluorescent intensities (Figures 4C and 4D). However, the rain did not affect the accuracy of CNV values even at low copy numbers (Figure 4E). Therefore, while it is advantageous to add the restriction enzyme to reduce error, the assay can still give accurate copy number even without restriction digestion, demonstrating the robustness of the assay in the extreme conditions.

### Comparison of variation between cell reference and hybrid amplicon

**Figure 5:**
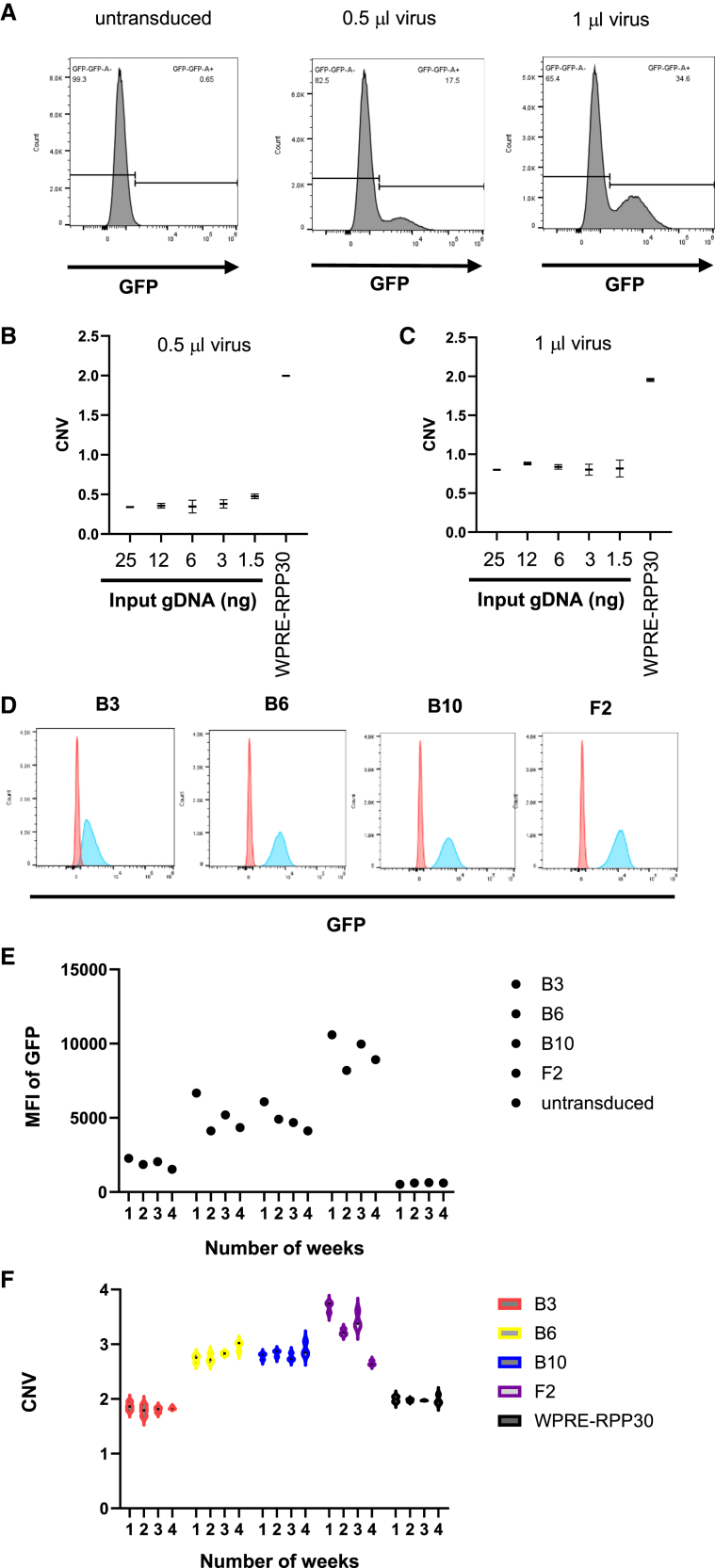
Validation of VCN assay with cell reference and hybrid amplicon (A) Jurkat cells were transduced with lentivirus expressing anti-CD19 CAR.P2A.GFP with 0.5 μL and 1 μL of viruses. GFP expression was measured with flow cytometry. (B and C) gDNA was extracted from the transduced cells. The *WPRE*-*RPP30* VCN assay was performed in ddPCR at different inputs of gDNA. Each condition was performed in triplicates. The mean values of triplicates with standard deviation as errors were plotted. (D) Jurkat cells were transduced with 3 μL of virus, and each clone of the transduced cells was isolated in the single cell mode. GFP expression histograms of the clones (blue) and untransduced cells (red) are overlayed. The clones were cultured for 4 weeks. Every week, the cells were measured for GFP expression with flow cytometry, and gDNA of the cells were extracted. (E) Median fluorescent intensity (MFI) values of GFP expression were plotted for each week. (F) Copy number of *WPRE*/*RPP30* of each clone was measured with ddPCR and plotted in a violin plot in GraphPad Prism for each week. Each condition was performed in triplicates. The bars indicate the variation in CNV values.

Ultimately, cell reference standards need to be established to monitor the assay variability throughout the life cycle of cell therapy development. However, establishing cell standards takes significant time and resources for development and validation. In addition, the previous study showed that the clones of cell standards varied when the cells are maintained for several weeks.^18^ To establish cell standards for chimeric antigen receptor (CAR)-expressing cells, Jurkat cells were infected with lentivirus expressing anti-CD19 CAR with green fluorescent protein (GFP). The cells were infected with different amounts of virus until the expression level of GFP is 10%–30% (Figure 5A). The WPRE/RPP30 assay was performed at different input levels of gDNA of the transduced cells. Indeed, the assay could determine the VCN of cells infected with lentivirus accurately as low as 1.56 ng of gDNA (Figures 5B and 5C). The results confirm that the assay detects both low copies of hybrid amplicons and the gDNA of transduced cells.

To establish the reference cell standards, single cell sorting was performed on the transduced cells. Clones were screened for different expression levels. Four clones with varying expression levels were selected for measuring assay variability (Figure 5D). GFP expression levels of those four clones were measured weekly with flow cytometry. Median fluorescent intensity (MFI) of GFP was relatively stable for B3, a clone with low expression of GFP. Higher variability was observed in clones expressing higher levels of GFP (Figure 5E).

gDNA of the transduced clones was extracted every week to determine copy numbers of *WPRE* and *RPP30*. Copy-number variation assays of *WPRE* and *RPP30* were performed from gDNA of those transduced clones together with *WPRE*-*RPP30* hybrid amplicons. As expected, the gDNA of the clones showed similar variabilities compared to the hybrid amplicons at low CNV numbers (Figure 5F). The results indicate that although cell standards are essential, they may not differentiate between biological and assay variabilities. In addition, we also observed that the cell standards showed lower day-to-day variability at lower copy numbers (Figure 5F). This observation has been observed in a previous study.^18^ In conclusion, the hybrid amplicons can accurately predict assay variability and offer unique advantages for assay development.

## Discussion

Over a hundred modalities involving cell and gene therapy have been approved through the end of the first quarter of 2023.^19^ Standardizing procedures to harmonize the analytical assays have become increasingly necessary to ensure accuracy and consistency of data.^20^ Efforts to harmonize the flow cytometry assays have been pursued and established. Internal QC beads have been developed to track the qualification status of the flow cytometry instruments. These recent developments provide users and data reviewers confidence in the results generated by the flow cytometry instruments. Daily QC beads are measured, and Levy Jennings plots are typically generated to monitor the performance of the instruments. These tracking methods ensure the validity of the data and the status of the instruments. If the tracking is out of specification, the instruments require maintenance services such as laser alignment. These activities ensure harmonization of data regardless of the environment and operators.

To date, standard harmonization methods to verify the performance status of dPCR instruments have not been developed or widely accepted. dPCR instruments are typically qualified by vendors but monitoring systems on day-to-day activities are left for end users to pursue independently. Commercial reference materials to check internal QC standards for dPCR instruments are currently not available. Moreover, operational qualification procedures do not include measuring droplet volumes or methods to confirm accuracy of measurement. A previous study measured the droplet volume variation longitudinally over 10 months and observed variations over two standard deviations, leading to bias in measuring nucleic acid concentrations.^21^ The study also observed that the measured volume was significantly lower than the manufacturer’s specified volume, suggesting independent verification of droplet reactions is critical. Since techniques to measure the droplet volumes are not easily assessable in typical labs, a qualification method with the commercially available reference materials is warranted.

An ideal reference standard for a VCN assay is the cells infected with known copy number of lentivirus. In 2021, the National Institute of Standards and Technology and Lentigen partnered to create Jurkat cell clones with VCN 1, 2, 3, and 4.^18^ VCNs of these cells were confirmed via NGS sequencing for exact copy numbers.^18^ However, researchers who adopt this approach require significant resources and capital investment to create these cell lines. They also require significant effort to keep the cells in culture and prevent genetic drift from occurring. In addition, these cancer cells may not be representative of the primary samples and create lot-to-lot variability. Here, we also observed that variability is higher when the cells are infected in multiple copies of transduced genes.

Therefore, commercially available reference materials are an attractive solution, as they are produced in controlled environments. Three types of reference standards recommended for assay validation, according to the US Food and Drug Administration (FDA) and European Medicines Agency (EMA), include (1) certified reference standards, (2) commercially available reference materials, and (3) materials documented for purity in report.^22^ gBlocks are an example of this type of reference material. However, as mentioned in previous publication,^22^ this type of reference material relies on the concentrations provided by the commercial suppliers. The hybrid amplicon material may fall into the second category of reference materials since it can be generated by commercial suppliers. Since reference amplicon was added to every strand, the concern over inconsistency in concentration is eliminated.

The hybrid amplicon approach can be extended beyond a simple duplex amplicon. The current DNA synthesis allows limiting the copy number to two because the commercial supplier does not allow redundant copies of DNA into the synthesis. Therefore, only one-to-one ratio of *WPRE* and RPP30 can be added. If DNA can be synthesized to circumvent redundancy, one could theoretically synthesize 2*WPRE*:1*RPP30*, 3*WPRE*:1*RPP30*, and 4*WPRE*:1*RPP30* amplicons. In this manner, linearity of copy numbers can be established.

In addition to using duplex DNA, one could also add amplicons of one or more different genes to test multiplex ddPCR assays. A common diagnostic application of these assays is detecting single nucleotide polymorphisms (SNPs). The human genome is highly diverse and variable, with any two humans differing on average at about 1 in every 1,000 DNA base pairs. Some SNPs lead to disease phenotypes, necessitating the need to develop diagnostic assays that properly identify genomic variations.^23^ SNP assays can detect changes on a single nucleotide scale. However, to characterize SNP assays, it is necessary to develop positive controls. Designing several amplicons of SNPs of the same gene linked together by reference amplicon offers an ideal strategy to ensure accuracy and specificity of SNP multiplex PCR assays. In addition, multiplex amplicon can also be used to develop assays to characterize complex viral vectors such as adeno-associated virus (AAV). In characterizing AAVs, it is important to determine the genomic integrity of viral particle where different components of AAV genomes can be linked together.^24^ Genomic integrity can be modeled after the hybrid amplicons linking different components of AAV genomes. In addition, it is possible to use hybrid amplicons as a positive control for mile-post analysis of two linked genes in the same chromosomes. Mile-post analysis can reveal how far apart the two linked genes in the chromosomes are.^25^ In summary, the use of hybrid amplicons is an essential tool for developing and characterizing a wide range of analytical PCR assays.

## Materials and methods

### Synthesis of reference materials

gBlock gene fragments used in this study were synthesized from Integrated DNA Technologies.

*WPRE* gBlock sequence-

5′-AATCAACCTCTGGATTACAAAATTTGTGAAAGATTGACTGGTATTCTTAACTATGTT-GCTCCTTTTACGCTATGTGGATACGCTGCTTTAATGCCTTTGTATCATGCTATTGCTTCCCGTATGGCTTTCATTTTCTCCTCCTTGTATAAATCCTGGTTGCTGTCTCTTTATGAGGAGTTGTGGCCCGTTGTCAGGCAACGTGGCGTGGTGTGCACTGTGTTTGCTGACGCAACCCCCACTGGTTGGGGCATTGCCACCACCTGTCAGCTCCTTTCCGGGACTTTCGCTTTCCCCCTCCCTATTGCCACGGCGGAACTCATCGCCGCCTGCCTTGCCCGCTGCTGGACAGGGGCTCGGCTGTTGGGCACTGACAATTCCGTGGTGTTGTCGGGGAAGCTGACGTCCTTTCCATGGCTGCTCGCCTGTGTTGCCACCTGGATTCTGCGCGGGACGTCCTTCTGCTACGTCCCTTCGGCCCTCAATCCAGCGGACCTTCCTTCCCGCGGCCTGCTGCCGGCTCTGCGGCCTCTTCCGCGTCTTCGCCTTCGCCCTCAGACGAGTCGGATCTCCCTTTGGGCCGCCTCCCCGCCTG-3'

*WPRE*-*RPP30* gBlock sequence-

5′-TGGCCCGTTGTCAGGCAACGTGGCGTGGTGTGCACTGTGTTTGCTGACGCAAC-CCCCACTGGTTGGGGCATTGCCACCACCTGTCAGCTCCTTTAAGCTTTCGGCCATCAGAAGGAGATGAAGATTGTCTTCCAGCTTCCAAGAAAGCCAAGTGTGAGGGCTGAAAAGAATGCCCCAGTCTCTGTCAGCACTCCCTTCTTCCCTTTTATAGTTCATCAGCCAC-3′

### Lentivirus preparation

pLV-EF1A-CD19.8H.8TM.BB.3z, anti-CD19 chimeric antigen receptor in lentiviral backbone, was described before.^26^ The plasmid was purchased from addgene. The viral packaging procedure is based on the four-plasmid system as the packaging plasmids were synthesized and provided by Carrigent, Inc. The 293-T-suspension-adapted cells were transfected with packaging plasmids using the PEI MAX method.

### Lentivirus transduction

Jurkat cells were purchased from ATCC. Jurkat cells were cultured and maintained in RP10 (RPMI medium with 10% fetal bovine serum, 1 mM sodium pyruvate, 2 mM GlutaMax with penicillin and streptomycin); 200,000 Jurkat cells were incubated with lentivirus in 6 μg/mL of polybrene in RP10 medium. The cells were centrifuged at 757×g for 1 h at 32°C. The cells were measured for GFP expression with Attune NxT flow cytometer (Thermo Fisher Scientific).

### Single cell sorting

GFP+ Jurkat cells were sorted with Wolf G2 Cell Sorter (NanoCellect) in single cell dispensing mode into 96-well plate. After sorting, the cells were incubated at 37°C. When single cell clones grew up, the cells were screened for GFP expression.

### Isolation of genomic DNA

gDNA was isolated using QIAamp DNA mini kit (Qiagen) according to the manufacturer’s instructions. gDNA was eluted and diluted in nuclease free water.

### Droplet digital PCR assay

The hybrid amplicon gene fragments were resuspended according to manufacturing protocol at 10 ng/mL in IDTE, according to the manufacturer’s certificate of analysis. Briefly, the tube containing gBlocks was centrifuged at 3,000×g. The IDTE was added to the tube to resuspend the gBlocks at 10 ng/mL. The fragments were serially diluted by 10-fold in IDTE. The diluted gBlocks in IDTE were further diluted in nuclease-free water by 7.7-fold. PCR reactions were prepared in Air Clean 600 PCR workstation. Twenty-two microliters of ddPCR reactions were prepared with a master mix containing 2× ddPCR Supermix for Probes without dUTP, WPRE custom assay (FAM), RPP30 validated assay (HEX), and HindIII (2,000 U/mL). Primers and probe for WPRE have been described previously.^15^ The WPRE custom assay for ddPCR was designed according to Bio-Rad’s specifications at primer and probe concentrations of 900 nM and 250 nM, respectively. RPP30 assay (10031244, Bio-Rad) has been developed and validated by Bio-Rad. HindIII was purchased from New England Biolabs. Master mix with gBlocks were prepared, and droplets were generated by Auto Droplet Generator (Bio-Rad). The plate was sealed after droplets were generated. The PCR was performed in C1000 thermal cycler (Bio-Rad) according to the cycling conditions shown in Table S1. At the end of the PCR cycle, droplets were read with QX200 droplet reader (Bio-Rad).

## Data availability

The authors confirm that the data supporting the findings of this study are available within the article and its supplemental information. All requests for raw and analyzed data and materials are available from Raymond Wu (raymondwu@sacfamerica.org) upon reasonable request.

## Acknowledgments

This study was partially funded by Sino-American Cancer Foundation. The authors would like to thank John Nguyen for helpful discussion and suggestions.

## Author contributions

R.W. conceptualized, designed, conducted experiments, and interpreted results. R.W. and F.L. wrote the manuscript. S.K. produced the lentivirus. Y.Y. provided advice and funding.

## Declaration of interests

The authors declare no competing interests.

## Supplemental information

Document S1. Table S1

Document S2. Article plus supplemental information

## References

[1] Morley A.A. (2014). Digital PCR: A brief history. Biomol. Detect. Quantif..

[2] Huggett J.F., Foy C.A., Benes V., Emslie K., Garson J.A., Haynes R., Hellemans J., Kubista M., Mueller R.D., Nolan T. (2013). The digital MIQE guidelines: Minimum Information for Publication of Quantitative Digital PCR Experiments. Clin. Chem..

[3] Basu A.S. (2017). Digital Assays Part I: Partitioning Statistics and Digital PCR. SLAS Technol..

[4] Sanders R., Huggett J.F., Bushell C.A., Cowen S., Scott D.J., Foy C.A. (2011). Evaluation of digital PCR for absolute DNA quantification. Anal. Chem..

[5] Gao S., Xu T., Wu L., Zhu X., Wang X., Chen Y., Li G., Li X. (2024). Complete Prevention of Bubbles in a PDMS-Based Digital PCR Chip with a Multifunction Cavity. Biosensors (Basel).

[6] Košir A.B., Divieto C., Pavšič J., Pavarelli S., Dobnik D., Dreo T., Bellotti R., Sassi M.P., Žel J. (2017). Droplet volume variability as a critical factor for accuracy of absolute quantification using droplet digital PCR. Anal. Bioanal. Chem..

[7] Majumdar N., Banerjee S., Pallas M., Wessel T., Hegerich P. (2017). Poisson Plus Quantification for Digital PCR Systems. Sci. Rep..

[8] Dong L., Meng Y., Sui Z., Wang J., Wu L., Fu B. (2015). Comparison of four digital PCR platforms for accurate quantification of DNA copy number of a certified plasmid DNA reference material. Sci. Rep..

[9] Martinez-Fernandez de la Camara C., McClements M.E., MacLaren R.E. (2021). Accurate Quantification of AAV Vector Genomes by Quantitative PCR. Genes.

[10] Dong L., Yoo H.-B., Wang J., Park S.-R. (2016). Accurate quantification of supercoiled DNA by digital PCR. Sci. Rep..

[11] Blackburn J., Wong T., Madala B.S., Barker C., Hardwick S.A., Reis A.L.M., Deveson I.W., Mercer T.R. (2019). Use of synthetic DNA spike-in controls (sequins) for human genome sequencing. Nat. Protoc..

[12] Whale A.S., Cowen S., Foy C.A., Huggett J.F. (2013). Methods for applying accurate digital PCR analysis on low copy DNA samples. PLoS One.

[13] Donello J.E., Loeb J.E., Hope T.J. (1998). Woodchuck hepatitis virus contains a tripartite posttranscriptional regulatory element. J. Virol..

[14] Ma J., Shao L., Fuksenko T., Liu H., Shi R., Dinh A., Highfill S.L., Zhang N., Panch S.R., Somerville R.P. (2023). Reference gene selection for clinical chimeric antigen receptor T-cell product vector copy number assays. Cytotherapy.

[15] Lizée G., Aerts J.L., Gonzales M.I., Chinnasamy N., Morgan R.A., Topalian S.L. (2003). Real-time quantitative reverse transcriptase-polymerase chain reaction as a method for determining lentiviral vector titers and measuring transgene expression. Hum. Gene Ther..

[16] Bustin S.A., Benes V., Garson J.A., Hellemans J., Huggett J., Kubista M., Mueller R., Nolan T., Pfaffl M.W., Shipley G.L. (2011). Primer sequence disclosure: a clarification of the MIQE guidelines. Clin. Chem..

[17] Russo J., Sheriff F., de Cicco R.L., Pogash T.J., Nguyen T., Russo I.H., Russo J., Russo I.H. (2014). Techniques and Methodological Approaches in Breast Cancer Research.

[18] Paugh B.S., Baranyi L., Roy A., He H.-J., Harris L., Cole K.D., Artlip M., Raimund C., Langan P.S., Jana S. (2021). Reference standards for accurate validation and optimization of assays that determine integrated lentiviral vector copy number in transduced cells. Sci. Rep..

[19] Chancellor D., Barrett D., Nguyen-Jatkoe L., Millington S., Eckhardt F. (2023). The state of cell and gene therapy in 2023. Mol. Ther..

[20] Le Lann L., Jouve P.-E., Alarcón-Riquelme M., Jamin C., Pers J.-O., PRECISESADS Flow Cytometry Study Group, PRECISESADS Clinical Consortium (2020). Standardization procedure for flow cytometry data harmonization in prospective multicenter studies. Sci. Rep..

[21] Emslie K.R., H McLaughlin J.L., Griffiths K., Forbes-Smith M., Pinheiro L.B., Burke D.G. (2019). Droplet Volume Variability and Impact on Digital PCR Copy Number Concentration Measurements. Anal. Chem..

[22] Bower J.F., McClung J.B., Watson C., Osumi T., Pastre K. (2014). Recommendations and best practices for reference standards and reagents used in bioanalytical method validation. AAPS J..

[23] Shastry B.S. (2007). SNPs in disease gene mapping, medicinal drug development and evolution. J. Hum. Genet..

[24] Prantner A., Maar D. (2023). Genome concentration, characterization, and integrity analysis of recombinant adeno-associated viral vectors using droplet digital PCR. PLoS One.

[25] Manderstedt E., Lind-Halldén C., Ljung R., Astermark J., Halldén C. (2020). Detection of F8 int22h inversions using digital droplet PCR and mile-post assays. J. Thromb. Haemostasis.

[26] Timpanaro A., Piccand C., Dzhumashev D., Anton-Joseph S., Robbi A., Moser J., Rössler J., Bernasconi M. (2023). CD276-CAR T cells and Dual-CAR T cells targeting CD276/FGFR4 promote rhabdomyosarcoma clearance in orthotopic mouse models. J. Exp. Clin. Cancer Res..

