# Supplementary material for: A method to validate viral copy-number assay involving a hybrid amplicon and duplex droplet digital PCR
Source: Mol Ther Methods Clin Dev. 2025 Apr 30;33(2):101483. doi: 10.1016/j.omtm.2025.101483 (PMC12229726; doi:10.1016/j.omtm.2025.101483)
Supplement: Document S1. Table S1 [file mmc1.pdf]

OMTM, Volume *33*

Supplemental information

A method to validate viral copy-number assay involving a hybrid amplicon and duplex droplet digital PCR

Raymond Wu, Frank Luh, Soo-Mi Kweon, and Yun Yen

Table S1: PCR cycling condition used in the experiment.

**Cycling Step**	**Temperature (°C)**	**Time (min)**	**Ramp Rate (°C/sec)**	**# Cycles**
Enzyme activation	95	10	2°C/sec	1
Denaturation	94	0.5		40
Annealing/extension	60	1		
Enzyme deactivation	98	10		1
Cool down	4	5	1°C/sec	1
Ambient temperature	25	infinite	1°C/sec	1

*Use a heated lid set to 105 °C and set the sample volume to 40 μl.
